# How Affective States Moderate the Relationship of Pain Catastrophizing With Pain Severity in Patients With Acute and Chronic Pain

**DOI:** 10.1002/brb3.71820

**Published:** 2026-10-13

**Authors:** Quinte T. Y. Kuper, Jasmijn E. Willemen, Richel Lousberg, Ruud J. M. Severeijns, Therese A. M. J. van Amelsvoort, Andrea J. R. Balthasar

**Affiliations:** ^1^ School for Mental Health and Neuroscience Maastricht the Netherlands; ^2^ Department of Emergency Medicine Laurentius Hospital Roermond Roermond the Netherlands; ^3^ Department of Psychiatry and Psychology MUMC+ Maastricht the Netherlands; ^4^ Department of Anesthesiology and Pain Medicine MUMC+ Maastricht the Netherlands

**Keywords:** acute pain, affect, chronic pain, experience sampling method, multilevel modeling, pain catastrophizing

## Abstract

**Background:**

Psychological factors, including mood, affect, and pain catastrophizing, critically influence pain perception. We aimed to examine the relationships of these factors with pain trajectories in both acute postsurgical pain (APSP) and chronic non‐cancer pain (CNCP) cohorts.

**Methods:**

We included 31 patients who underwent total knee arthroplasty (TKA) and 21 patients with CNCP. All included patients completed the pain catastrophizing scale (PCS) questionnaire. In addition, patients reported their status within their natural environment (ten times a day for 6 days) using an experience sampling method—a smartphone app, which contains items regarding pain, positive affect (PA), and negative affect (NA).

**Results:**

Pain scores showed a normal distribution in both cohorts; the APSP cohort reported significantly lower overall pain scores than the CNCP cohort (*p = *0.003). In both cohorts, affective state was significantly associated with pain severity (*p < *0.001). However, PCS scores were not significantly associated with pain severity. Notably, a higher PA was associated with lower pain severity among patients with high PCS scores, with a stronger association in the APSP cohort.

**Conclusions:**

Our findings indicate that affective state is associated with pain severity in both APSP and CNCP cohorts. The combination of low PA and high PCS may be relevant for identifying individuals with higher pain severity, particularly in the APSP cohort. These results provide a basis for future research on targeted approaches aimed at enhancing PA and reducing catastrophic pain‐related thoughts.

**Statement of significance:**

Our study shows that affective states play a key role in pain severity in both acute and chronic pain cohorts. Positive affect is associated with lower pain severity in patients with high PCS scores, with a stronger effect after surgery than in chronic pain. These results suggest that early identification of high catastrophizing and low positive affect may support targeted interventions to enhance recovery and potentially reduce the risk of developing chronic pain.

## Graphical abstract

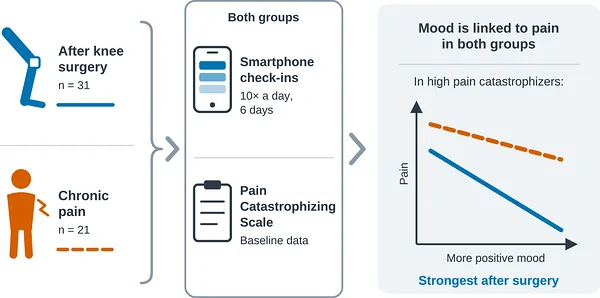

## 1 Introduction

Pain is a complex, multifaceted experience that affects an individual's quality of life. It can be broadly categorized as acute and chronic pain. Acute pain, especially acute postsurgical pain (APSP), necessitates effective pain management strategies to facilitate recovery and prevent complications such as pain chronification (Kehlet and Dahl 2003; Wylde et al. 2011). Chronic non‐cancer pain (CNCP), which is defined as pain persisting for >3 months, has a substantial prevalence and poses a significant burden on individuals and healthcare systems (Breivik et al. 2006; Johannes et al. 2010). In addition, CNCP is associated with various adverse outcomes, including reduced physical functioning, psychological distress, and diminished quality of life (Smith et al. 2001). Notably, inadequate management of APSP can lead to sensitization of the nervous system, which causes pain chronification (Chapman and Vierck 2017). Therefore, it is important to elucidate the factors underlying postsurgical pain chronification in order to inform preventive interventions.

In 2023, there were ≈40,000 cases of total knee arthroplasty (TKA) in the Netherlands, with 20% of them involving a risk of CNCP (LROI 2025). A UK study emphasizes that the cost of pain after TKA for the NHS was >£10 million/annum (Kassam et al. 2012), which further suggests the need to elucidate factors related to APSP and CNCP.

Psychological factors, including mood and affect, critically influence pain perception. Mood refers to a prolonged emotional state that influences a person's general feeling, while affect refers to the outward and more dynamic expression of that emotion (Manjunatha et al. 2009). Affect can be categorized as positive affect (PA; including cheerfulness, relaxation, and satisfaction) or negative affect (NA; including anger, anxiety, and irritation); further, it can influence pain perception (Watson and Clark 1994).

NA levels are positively associated with pain sensitivity and intensity, while PA may have analgesic effects and promote pain resilience (Finan et al. 2013; Hanssen et al. 2013). There has been extensive research on the influence of NA on pain; however, the role of PA remains unclear (Finan and Garland 2015; Hanssen et al. 2017; Ong et al. 2020; Ong et al. 2010; Sobol‐Kwapinska et al. 2016). Nonetheless, higher PA levels have been associated with reduced pain and improved adjustment in patients with chronic pain; however, these relationships are mediated by cognitive–emotional factors, including pain catastrophizing and coping styles (Hanssen et al. 2017; Finan and Garland 2015; Ong et al. 2020).

Pain catastrophizing, which is characterized by magnification of pain‐related thoughts, rumination, and feelings of helplessness in relation to pain, significantly influences pain perception and the risk of chronic pain (Balthasar et al. 2023; Simic et al. 2024; Sullivan and Pivik 1995; Wertli et al. 2014). High levels of pain catastrophizing are associated with higher pain intensity, greater disability, and poorer treatment outcomes in both patients with APSP and CNCP (Burns et al. 2012; Edwards et al. 2004). Among patients undergoing TKA, pain catastrophizing is a significant predictor of prolonged postsurgical pain (Riddle et al. 2010). Taken together, it is important to elucidate the interplay among PA/NA, pain catastrophizing, and pain perception in order to develop comprehensive psychological profiles and personalized treatments.

Some patients with APSP may develop chronic pain after TKA. Recent studies using the experience sampling method, which allows collection of real‐time data regarding pain and psychological factors, have provided valuable insights into real‐time fluctuations of pain and affective states (Balthasar et al. 2023; van Os et al. 2017).

This study aimed to investigate how affective states moderate the relationship of pain catastrophizing with pain severity in APSP and CNCP populations. The results may help to generate hypotheses for future intervention studies targeting emotional and cognitive factors involved in pain experiences across different pain conditions.

## 2 Methods

### 2.1 Study Design and Participants

This study included two distinct cohorts. The APSP cohort comprised participants who experienced APSP following TKA at Maastricht University Medical Centre+, the Netherlands (data collection period: December 2017–March 2019). The CNCP cohort comprised participants with CNCP from the outpatient pain clinic at Maastricht University Medical Centre+ (MUMC+), Maastricht, the Netherlands (data collection period: from June 7, 2019 to November 10, 2020).

### 2.2 Ethical Considerations

This article reports a secondary analysis of data from two observational cohort studies conducted in accordance with the Declaration of Helsinki and approved by a Medical Research Ethics Committee (METC; reference numbers 2017‐0145 and 2018‐0955 for the APSP and CNCP cohorts, respectively). All participants provided written informed consent, and data confidentiality and participant anonymity were maintained throughout the study. Data from both cohorts have been reported previously (Balthasar et al. 2023; Kuper et al. 2025). The present manuscript addresses a distinct research question by examining whether affective states moderate the relationship between pain catastrophizing and pain severity across both cohorts and was conducted within the scope of the original METC approvals and informed consent procedures.

### 2.3 Inclusion and Exclusion Criteria

For the APSP cohort, the inclusion criteria were age ≥ 18 years, having undergone primary TKA surgery, ability to provide informed consent, having a smartphone compatible with the ESM app, and proficiency in Dutch. The exclusion criteria included cognitive impairments or conditions interfering with the ability to complete ESM assessments and having been admitted to the hospital.

For the CNCP cohort, the inclusion criteria were age ≥ 18 years, having chronic pain lasting ≥ 12 weeks, ability to provide informed consent, having a smartphone compatible with the ESM app, and proficiency in Dutch. The exclusion criteria were similar to those for the APSP cohort.

### 2.4 Data Collection

In both studies, data were collected using the Psymate application (www.psymate.eu), which is a digital ESM tool that was validated in 2017 (van Os et al. 2017). The Psymate application allows real‐time data collection and thus reduces recall bias.

The data collection and ESM procedure in both cohorts (APSP and CNCP) is summarized in Figure 1. Participants received 10 semi‐random acoustic alerts per day over 6 consecutive days.  For the CNCP cohort, data collection started prior to the initial physician appointment. The CNCP cohort comprised participants with CNCP from the outpatient pain clinic at Maastricht University Medical Centre+ (MUMC+), Maastricht, the Netherlands (data collection period: from June 7, 2019 to November 10, 2020), with chronic pain complaints lasting at least 12 weeks. ESM assessment started before the first pain clinic consultation; therefore, inclusion was based on referral for chronic pain and pain duration rather than on a final pain clinic diagnosis established during that consultation. Participants were not classified according to chronic primary versus chronic secondary pain. To ensure between‐cohort comparability, CNCP participants enrolled during the COVID‐19 pandemic were excluded. This decision was based on previously published analyses of the CNCP cohort showing that, although baseline patient characteristics did not significantly differ between the pre‐pandemic and pandemic groups, COVID‐19 status significantly moderated the relationship between positive affect and daily pain trajectories (Kuper et al. 2025). The APSP cohort comprised patients with knee arthritis undergoing TKA, representing a chronic pain population experiencing APSP following surgery. Baseline measurements, including the Pain Catastrophizing Scale (PCS), were obtained preoperatively, whereas ESM data were collected postoperatively. For all APSP participants, the postoperative ESM assessment period commenced upon hospital discharge and followed the same standardized protocol of 10 semi‐random assessments per day over 6 consecutive days. Therefore, only ESM data obtained after hospital discharge were included for the APSP cohort, ensuring comparability between cohorts. Alerts were evenly distributed between 7:30 a.m. and 10:30 p.m. to ensure comprehensive coverage of the waking day and minimize anticipation effects. Each alert prompted participants to complete several items regarding current pain levels and affective states (PA/NA).

**FIGURE 1:**
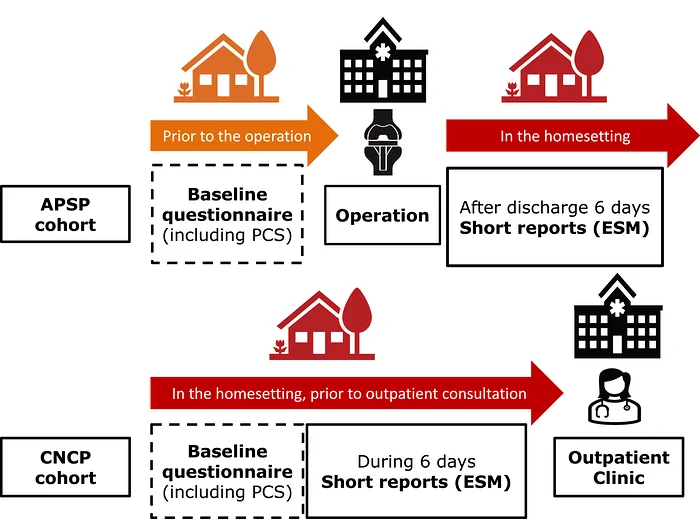
Study design and ESM data collection procedure in APSP and CNCP cohorts.

Participants were instructed to maintain smartphone notifications to avoid missing alerts. Responses were considered valid only if completed within 15 min of the alert. In addition, participants were required to complete a minimum of 18 reports (30% of the maximum 60 prompts) overall for inclusion in the analysis in order to ensure adequate statistical power and data reliability (Delespaul 1995). Participants received technical support throughout the data collection period, as needed.

### 2.5 Pain Variables

Pain severity in the CNCP and APSP cohorts was assessed using a 0–10 and 1–7 numeric rating scale, respectively. To ensure between‐cohort comparability, pain intensity scores were normalized to a 0–100 scale using min–max transformation: ((rating − minimum possible score) / (maximum possible score − minimum possible score)) × 100. Thus, for the APSP 1–7 scale, a score of 1 corresponded to 0%, and a score of 7%–100%, with intermediate values proportionally scaled. For the CNCP 0–10 scale, 0 corresponded to 0%, and 10%–100%.

### 2.6 Pain Catastrophizing

The PCS is a validated 15‐item self‐assessment questionnaire for assessing rumination, magnification, and helplessness related to pain experiences (pain catastrophizing), with the total score ranging from 0 to 55. Higher PCS scores (> 30) indicate greater levels of pain catastrophizing (Darnall et al. 2017).

### 2.7 Affect

Regarding affective states, participants rated their current emotional states on a 1–7 numeric rating scale (1 = “not at all,” 7 = “very much”). The Dutch term for each affect is provided in parentheses. The PA items included cheerful (“opgewekt”), relaxed (“ontspannen”), and satisfied (“tevreden”), whereas the NA items included irritated (“geïrriteerd”), guilty (“schuldig”), lonely (“eenzaam”), worry (“piekeren”), and anxious (“angstig”) (Watson and Clark 1994). For each ESM assessment (Table S1), the PA items were averaged to create one composite PA score, and the NA items were averaged to create one composite NA score. These composite PA and NA scores were entered into the multilevel models as raw time‐varying predictors. No person‐mean centering or decomposition into within‐person and between‐person components was performed. Therefore, the estimated effects reflect a combination of within‐person fluctuations and between‐participant differences in affective states.

### 2.8 Other Variables

Additional information, including body mass index (BMI), age, and sex, was obtained from the medical records.

### 2.9 Sample Size Calculation

A formal sample size calculation was not performed, as this was an observational, explorative study. Owing to the ESM, each included participant contributed a high density of repeated observations, thereby enhancing the precision and reliability of within person analyses. The data obtained are intended to inform effect size estimates and power calculations for future hypothesis‐driven studies.

### 2.10 Statistical Analysis

First, we performed descriptive analyses to assess the distribution of the variables and perform between‐cohort comparisons. Between‐cohort comparisons of continuous and categorical variables were performed using independent t‐tests and Chi‐square tests, respectively. In cases of low cell frequencies (< 5), Fisher's exact test was employed.

### 2.11 Multilevel Regression

We applied multilevel analyses since the repeated measurements (ESM reports) are nested within participants, which created dependencies in the data that cannot be adequately addressed by conventional regression techniques (Table S2). The multilevel framework enables proper handling of within‐participant correlations between sequential observations, while allowing for between‐participant variation. Further, it incorporated random intercepts to account for baseline between‐participant differences in pain. The analysis was structured as a two‐level model, with ESM observations (Level 1) nested within participants (Level 2). An AR‐1 covariance structure was used, with the assumption that ESM reports closer to each other had a better correlation than those further apart.

In the model, pain was the dependent variable, while sex, age, and BMI were covariates.

### 2.12 Analysis Structure

In the analyses, we assessed the distribution of pain variables; main effect of PA, NA, PCS, and cohort on pain; and interaction effect of affect, cohort, and PCS on pain.

### 2.13 Statistical Software

All statistical analyses were conducted using SPSS Statistics for Windows, version 28.0 (IBM Corp., Armonk, NY). Statistical significance was set at *p* ≤ 0.05.

## 3 Results

Figure 2 presents a flowchart illustrating the number of participants lost to follow‐up and the reasons for exclusion. To ensure between‐cohort comparability, we excluded patients in the CNCP cohort who were enrolled during the COVID‐19 pandemic. Psychiatric and medical comorbidities, medication use, sleep disturbance, and other chronic health conditions were not assessed in the same way across both cohorts. Therefore, these variables were not included as exclusion criteria or covariates in the comparative analyses. To maintain comparability between the APSP and CNCP cohorts, the multilevel analyses were adjusted for variables that were available in both cohorts: age, sex, and BMI.

**FIGURE 2:**
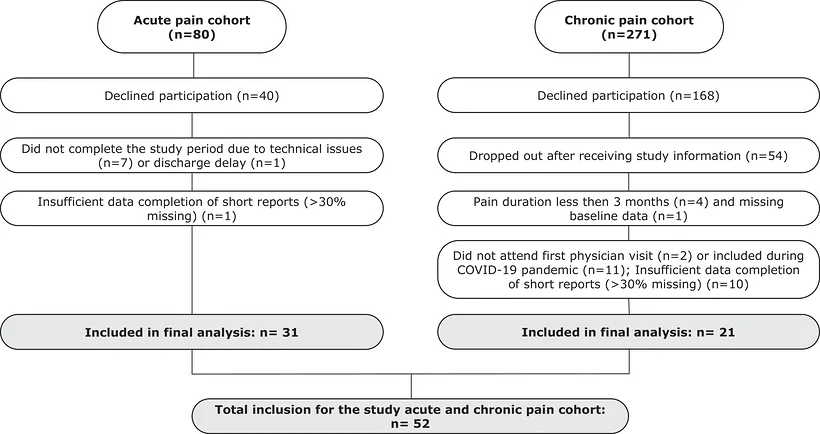
Flowchart of cohort inclusion.

### 3.1 Baseline Characteristics

Table 1 presents the baseline characteristics of both cohorts. Compared with the CNCP cohort, participants in the APSP cohort were significantly older and had a significantly higher BMI. No significant between‐cohort differences were observed in sex distribution or baseline PCS. In addition, patients in the CNCP cohort had different pain diagnoses, including neuropathic, nociceptive, and visceral pain. Further, patients in the APSP cohort underwent TKA for osteoarthritis with Kellgren–Lawrence Grade 2 and above, persistent pain, and functional limitation despite conservative therapy.

**TABLE 1:** Baseline patient characteristics of each cohort.

	APSP cohort	CNCP cohort
BMI^*^	29.65 ± 4.92	24.92 ± 4.59
Sex	female	female
Age^*^	61.53 ± 8.1	50.12 ± 12.42
PCS	24.06 ± 5.9	26.16 ± 11.39

*Note*: Values are presented as mean ± SD, unless otherwise indicated.

* Significant difference (*p < *0.05).

### 3.2 Main Effect of Pain

Before executing the multilevel regression analyses, the distribution of the dependent variable (pain score) was assessed. Pain scores were normally distributed (skewness = −0.196, kurtosis = −1.067) and no outlying values were observed.

### 3.3 Main Effect of NA, PA, PCS, and Cohort

Controlling for the aforementioned covariates, there was a significant (*p* < 0.001) main effect of NA and PA on pain. Specifically, each one‐point increase in NA and PA was associated with a 3.59‐ and 4.16‐point change in pain intensity on the 0–100 scale, respectively. There was a significant between‐cohort difference (*p = *0.003): pain severity was 26% lower in the APSP cohort than in the CNCP cohort. However, PCS did not show a significant effect on pain (*p = *0.09). In addition, there was a significant random intercept for baseline between‐participant differences in pain (*p* < 0.001). In the next step, NA, PA, PCS, and cohort were entered together to check for independence. Here, only the main effect of PA (*p* < 0.001) and cohort (*p = *0.001) remained significant.

### 3.4 Interaction Effects Between PA, PCS, and Cohort

We observed a significant second‐order PA × cohort interaction effect. Compared with the CNCP cohort, the APSP cohort exhibited a stronger negative association between PA and pain severity (Figure 2).

In a post hoc analysis, we assessed whether PCS moderated the second‐order PA × cohort interaction. A significant PA × cohort × PCS interaction emerged (Table 2). Figure 3 shows the effect of PA levels on pain at low (score = 15) and high (score = 35) PCS scores. As can be seen, pain intensity was differentially affected by the combination of PA levels and PCS scores in both cohorts. The strongest negative association between PA and pain severity was observed in the APSP cohort at the higher PCS level.

**TABLE 2:** Three‐way interaction between PA, PCS, and cohort.

3rd order interaction	Coefficient	Std. Error	*t*‐value	*p*‐value	95% CI LB	95% CI UB
PCS*PA*cohort	0.362	0.109	3.323	0.001	0.148	0.577

*Note*: The table presents the three‐way interaction between PA, PCS, and cohort. The complete multilevel model, including all main effects, lower‐order two‐way interactions, and the three‐way interaction, is presented in Table S2.

Abbreviations: CI, confidence interval; PA, positive affect; PCS, pain catastrophizing scale.

**FIGURE 3:**
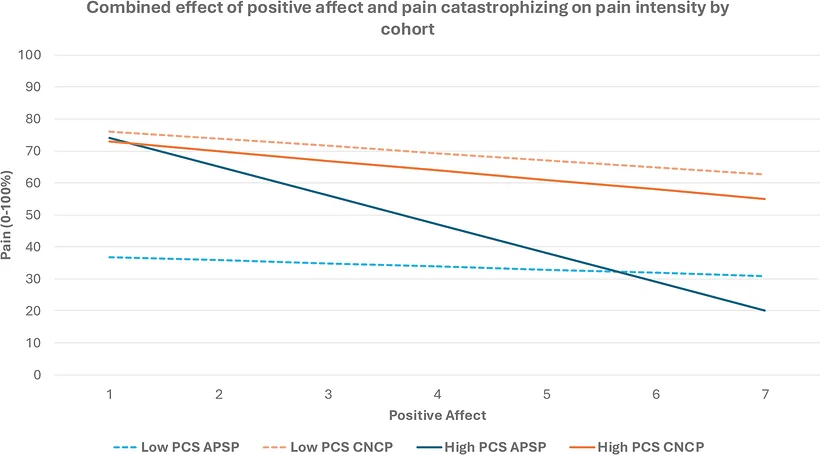
Model‐based predictions of the three‐way interaction between PA, PCS, and cohort. Lines represent predicted values derived from the multilevel model. For visualization, low and high PCS were set at illustrative values of 15 and 35, respectively; these values do not represent distinct participant categories.

## 4 Discussion

This study investigated how affective states moderate the relationship between pain catastrophizing and pain severity in APSP and CNCP populations. Consistent with aforementioned reports, higher PA levels were associated with lower pain. Notably, the association between PA and pain severity was stronger among patients with higher PCS scores, and was significantly more pronounced in the APSP cohort than in the CNCP cohort. This is consistent with previous reports regarding affect–pain relationships (Ong et al. 2010). However, the PCS score did not significantly influence the pain scores, which is inconsistent with previous reports (Hasegawa et al. 2022). This can be attributed to our pain assessment using the ESM tool, which extensively reduces recall bias, and our multilevel analysis, which accounts for different baseline levels of pain (random intercept). Both cohorts showed significant among‐patient differences in the baseline levels of pain. Among NA, PA, and PCS, only PA showed a significant, independent association with pain severity in the present models, regardless of NA and PCS scores. An additional consideration concerns the use of different pain rating scales across cohorts. Although pain intensity ratings were normalized to a common 0–100 scale using min‐max transformation, this approach does not establish psychometric equivalence between the original 1–7 and 0–10 scales. Therefore, between‐cohort comparisons and interaction effects based on the normalized pain scores should be interpreted with caution.

As aforementioned, the interaction between PA and PCS score was strongly associated with increased pain intensity, which indicated that the association between PA and pain severity differed according to the level of pain catastrophizing. Notably, this relationship was more pronounced in the APSP cohort than in the CNCP cohort. This further highlights the association between PA and pain severity in the context of higher levels of pain catastrophizing, particularly in the APSP cohort.

Contrastingly, there was no significant interaction between NA and PCS; furthermore, NA did not alter the PA × PCS interaction. This suggests that (low) PA levels may be more critical than high NA levels in shaping postsurgical pain trajectories. Although high NA levels are often associated with poor pain outcomes, our findings indicate that its unique contribution was modest with respect to the combined PA–PCS risk profile. Accordingly, preoperative screening for this risk profile could inform direct interventions for raising PA and reducing catastrophic thinking.

There are several hypotheses regarding the interaction between pain and affective states. Pain and emotion may share primitive neural pathways that evolved to promote protective survival behaviors (Gilam et al. 2020), with both overlapping and distinct conceptual and neuroanatomical areas. Maladaptive cognitive and emotional factors, including pain catastrophizing, have been linked to brain regions involved in pain processing, attention, emotion, motor activity, and reduced pain inhibition in chronic pain (Malfliet et al. 2017).

Neurobiological models may help explain why PA mitigated the impact of catastrophizing more strongly than NA exacerbated it. Ascending nociceptive signals are transmitted by different fiber types and pathways. Specifically, Aδ fibers travel via the lateral spinothalamic tract to the somatosensory cortices, coding sensory–discriminative features. Contrastingly, C fibers project through medial spinothalamic and spinoreticular pathways to the anterior cingulate cortex and insula, which mediate affective–motivational dimensions of pain (Apkarian et al. 2005). These affective circuits overlap with reward‐related networks, including the orbitofrontal cortex and nucleus accumbens, which are also involved in PA (Leknes and Tracey 2008). Activation of such systems can reduce pain unpleasantness and enhance descending inhibitory control via the periaqueductal gray and rostral ventromedial medulla. Contrastingly, NA is closely associated with increased amygdala activity, which may amplify pain and weaken inhibition. This framework suggests that insufficient PA levels may leave affective pain circuits unmodulated, while the presence of NA alone exerts a more modest, additive effect. Taken together, the neural architecture of pain supports our finding that low PA, especially when combined with high pain catastrophizing, yields a distinct risk profile for poor postoperative outcomes. These results suggest that both momentary affective‐pain dynamics and stable cognitive–emotional traits contribute to pain chronification, highlighting the importance of their combined influence.

To our knowledge, this is the first study on the influence of PA/NA/PCS on pain perception, measured using ESM, and involving multilevel analysis with random intercepts. Previous studies examining the relationship between pain and mood administered a daily short questionnaire (Cho et al. 2013; Feldman et al. 1999). Contrastingly, we used ESM as the assessment tool, which is a relatively novel approach (Balthasar et al. 2021).

Our findings suggest that the combination of low PA and high PCS is associated with higher pain severity, particularly in patients with APSP. This is consistent with previous reports showing that preoperative pain catastrophizing affects pain outcomes after TKA (Hasegawa et al. 2022).

Studies have also described distinct psychosocial subgroups among individuals with chronic pain, differing in distress, coping, and pain interference (Widerstrom‐Noga et al. 2007). This highlights the need for tailored interventions.

Accordingly, future studies could investigate whether preoperative assessment of PA and pain catastrophizing may help identify patients who could benefit from targeted interventions aimed at increasing PA and reducing catastrophic thoughts. These interventions include brief cognitive–behavioral counseling, mindfulness‐based approaches, or music listening (Pester et al. 2023; Schneider 2018).

This study has several strengths. The use of ESM allowed repeated assessments of pain and affect in participants’ daily lives, reducing recall bias and capturing fluctuations that may not be reflected in conventional retrospective questionnaires. This intensive longitudinal approach may provide insight into how affective states relate to pain severity in real‐world daily contexts. By distinguishing PA and NA, the study also allows a more nuanced examination of their differential associations with pain severity. In addition, the inclusion of both APSP and CNCP cohorts enabled exploration of affective‐cognitive associations across two clinically relevant pain contexts.

Several methodological considerations should be taken into account when interpreting the findings. PA and NA were entered as time‐varying predictors and were not decomposed into within‐person and between‐person components. Consequently, the observed associations reflect both momentary affective fluctuations and more stable between‐participant differences in affective functioning. This approach was consistent with the exploratory aim of the present study, while future studies may further clarify these findings by separating within‐person affective changes from between‐person differences.

Inclusion in the analyses required completion of a minimum of 30% of ESM reports, consistent with established ESM methodology. This may have influenced the representativeness of the final sample. However, applying a minimum ESM‐compliance criterion was necessary to support sufficient data quality and reliable estimation in the multilevel models. Similarly, the comparison between APSP and CNCP should be interpreted in light of the clinical and demographic differences between these cohorts. These differences reflect the distinct pain contexts under study and were inherent to the study objective, which was to examine whether associations between affect, pain catastrophizing, and pain severity differ according to pain context. Analyses were adjusted for age, sex, and BMI to account for key demographic differences between cohorts. Nevertheless, as in most observational studies, the potential influence of other clinical factors cannot be entirely ruled out.

Although no a priori sample size calculation was performed and the participant‐level sample size was modest, the ESM design yielded a rich dataset with repeated observations in daily life. This design allowed preliminary investigation of momentary affect‐pain associations and was consistent with the exploratory nature of the study. The observed PA × PCS × Cohort interaction was identified in a post hoc analysis and should therefore be interpreted as hypothesis‐generating. Nevertheless, this finding may indicate an affective–cognitive profile that is relevant to pain severity, particularly in the APSP cohort, and should be examined further in larger, prospectively designed studies.

Future studies should further examine within‐person affective dynamics and clarify the temporal direction of the associations between affect, catastrophizing, and pain. Longitudinal and interventional designs are needed to evaluate whether approaches aimed at enhancing PA and reducing catastrophic, pain‐related thoughts may improve pain‐related outcomes.

In conclusion, this study contributes to the growing literature on affective and cognitive factors in pain by examining these associations at the momentary level in daily life rather than relying solely on retrospective assessments. Using an ESM approach in both APSP and CNCP populations, the study explored whether the relationship between affect, pain catastrophizing, and pain severity differs across clinically distinct pain contexts. The findings suggest that low PA in combination with high PCS may be associated with higher pain severity, particularly in patients with APSP. These results highlight the potential importance of considering affective and cognitive factors jointly when studying pain experiences.

The observed pattern further suggests that the combination of low PA and high PCS scores may be clinically relevant for identifying individuals at risk of higher pain severity, particularly in the APSP cohort. This may inform future research on targeted interventions focusing on both enhancing PA and reducing catastrophic thinking.

## Author Contributions

**Quinte T.Y. Kuper**: conceptualization, formal analysis writing – original draft. **Jasmijn E. Willemen**: conceptualization, formal analysis writing – original draft. **Richel Lousberg**: methodology, formal analysis, writing. **Ruud J.M. Severeijns**: conceptualization, writing – original draft. **Therese A.M.J. van Amelsvoort**: writing – review and editing. All authors have read and agreed to the published version of the manuscript. **Andrea J.R. Balthasar**: conceptualization, formal analysis, data curation, supervision, writing – review and editing.

## Funding

The authors have nothing to report.

## Conflicts of Interest

The authors declare no conflicts of interest.

## Supporting information




**Supplementary Information**: brb371820‐sup‐0001‐SuppMat.pdf

**Supplementary Information**: brb371820‐sup‐0002‐SuppMat.doc

*Supplemental Table 1*: Translated ESM items used to assess positive affect and negative affect and pain.
*Supplemental Table 2*: Full multilevel interaction model with pain percentage as the dependent variable.

**Supplementary Information**: brb371820‐sup‐0004‐SuppMat.docx

## Data Availability

Data available on request from the corresponding author.
