# Supplementary material for: How Affective States Moderate the Relationship of Pain Catastrophizing With Pain Severity in Patients With Acute and Chronic Pain
Source: Brain Behav. 2026 Oct 13;16(10):e71820. doi: 10.1002/brb3.71820 (PMC13659935; doi:10.1002/brb3.71820)
Supplement: Supplementary file 1 [file BRB3-16-e71820-s001.pdf]

ELSEVIER

Certificate of Elsevier Language Editing Services

The following article was edited by Elsevier Language Editing Services:

Modulation of pain by different affective states in patients with a high pain catastrophizing score: acute versus chronic pain cohorts

Ordered by:

Andrea Balthasar

Estimated Delivery date:

2025-10-17

Order reference:

ASLEPLUS1116898
