# Supplementary material for: How Affective States Moderate the Relationship of Pain Catastrophizing With Pain Severity in Patients With Acute and Chronic Pain
Source: Brain Behav. 2026 Oct 13;16(10):e71820. doi: 10.1002/brb3.71820 (PMC13659935; doi:10.1002/brb3.71820)
Supplement: Supplementary file 2 [file BRB3-16-e71820-s003.docx]

**How affective states moderate the relationship of pain catastrophizing with pain severity in patients with acute and chronic pain**

| Article category | *Original article* |
| --- | --- |
| A running head | *Affect moderates pain perception in APSP and CNCP* |
| Name of Author(s) | *Quinte T.Y. Kuper^#^, Bsc^1^; Jasmijn E. Willemen^#^, Bsc^1^; Richel Lousberg, PhD^2^_;_ Ruud J.M. Severijns PhD^2^; Therese A.M.J. van Amersvoort, PhD^2^; Andrea J.R. Balthasar, PhD^3*^* |
| Affiliations | *^#Shared first authorship^*  *^1PhD candidate, School for Mental Health and Neuroscience, Maastricht, The Netherlands^*  *^2Department of Psychiatry and Psychology, MUMC+, Maastricht, The Netherlands^*  *^3Department of Anaesthesiology and Pain Medicine, MUMC+, Maastricht, The Netherlands^* |
| Corresponding Author | *Andrea J.R. Balthasar*  *Phone: +31 43 3874500*  *E-Mail:* [*a.balthasar@mumc.nl*](mailto:a.balthasar@mumc.nl) |
| Authors contributions | ☒ I have added a paragraph at the end of my manuscript (between the Discussion and the References).  *Example: This study was designed by A.A., B.B., and Z.Z. The experiments were performed by A.A., B.B., C.C., and D.D. The data were analyzed by A.A. and B.B., and the results were critically examined by all authors. A.A. had a primary role in preparing the manuscript, which was edited by Y.Y. and Z.Z. All authors have approved the ﬁnal version of the manuscript and agree to be accountable for all aspects of the work.* |
| Is this an observational study? | ☐ No  ☒ Yes  → Have you uploaded the STROBE checklist? ☒ Yes |
| Is this a systematic review? | ☒ No  ☐ Yes  　→ Have you uploaded the PRISMA checklist? ☐ Yes  　→ Registered in PROSPERO? ☐ (*Please provide the number*) |
| Is this an animal study? | ☒ No  ☐ Yes  　→ Have you included the statement that your study conforms to the guidelines such as ARRIVE? ☐ Yes |
| Plagiarism Declaration | ☒ We hereby declare that this paper is our own work, except where acknowledged, and has not been submitted elsewhere. |
| Public data depository | Data available on request from the author |
| Is the reviewer list appropriate? | ☒ Yes, we confirm that.  To avoid conflicts of interest, please suggest reviewers from different countries and with different nationalities. Please note that it is the authors’ responsibility to suggest appropriate reviewers. The failure to do so signifies that your work will not draw broad attention from readers and is susceptible to rejection. |
| Word count for the Introduction | *493 words, not exceeding 500 words* ***including the citations*** |
| Word count for the Discussion | *953 words, not exceeding 1500 words* ***including the citations*** |
| Number of references | *37 references, not exceeding 80 references for original articles*  *(120 for reviews, 6 for letters to the editor, 10 for case reports)* |
| Reference/citation styles comply with the authors’ guideline | ☒ Yes, we confirm that. |
| Number of Figures | *3* |
| Number of Tables | *2* |
| Funding sources (if applicable) | *This research received no external funding* |
| Any conflicts of interest? | *The authors declare no conflicts of interest* |
| Use of Artificial Intelligence | *Did you use generative artificial intelligence (AI) or AI-assisted technologies in the preparation of this manuscript?*  *If yes, please give the name of the tool(s) and how AI was used in your manuscript.* |
| Significance statement  *indicating the main aspects where this work adds significantly to existing knowledge in the field, and if appropriate to clinical practice.* | *69 words, not exceeding 80 words* |

*Note: Manuscripts not complying with the authors’ guideline will be returned without processing.*

[*https://onlinelibrary.wiley.com/page/journal/15322149/homepage/forauthors.html*](https://onlinelibrary.wiley.com/page/journal/15322149/homepage/forauthors.html)
