# Supplementary material for: How Affective States Moderate the Relationship of Pain Catastrophizing With Pain Severity in Patients With Acute and Chronic Pain
Source: Brain Behav. 2026 Oct 13;16(10):e71820. doi: 10.1002/brb3.71820 (PMC13659935; doi:10.1002/brb3.71820)
Supplement: Supplementary file 3 [file BRB3-16-e71820-s004.docx]

**Supplemental Table 1: ESM Questions Translated**

**Questions/Statements**

I feel cheerful

I feel relaxed

I feel irritated

I feel satisfied

I feel lonely

I feel anxious

I feel guilty

I feel worried

I have pain

ESM, Experience Sampling Method

**Supplemental Table 2: Results of Analyses**

| **Estimates of Fixed Effects^a^** | | | | | | | |
| --- | --- | --- | --- | --- | --- | --- | --- |
| Parameter | Estimate | Std. Error | df | t | Sig. | 95% Confidence Interval | |
|  |  |  |  |  |  | Lower Bound | Upper Bound |
| Intercept | 3,839871 | 29,784688 | 51,828 | 0,129 | 0,898 | -55,932194 | 63,611936 |
| BMI | 0,235968 | 0,603515 | 32,611 | 0,391 | 0,698 | -0,992450 | 1,464385 |
| age | -0,081553 | 0,266542 | 32,433 | -0,306 | 0,762 | -0,624198 | 0,461091 |
| Sex | -4,470740 | 5,684699 | 32,621 | -0,786 | 0,437 | -16,041454 | 7,099974 |
| cohort | 76,255279 | 24,133878 | 94,242 | 3,160 | 0,002 | 28,338502 | 124,172057 |
| PA | 5,010627 | 2,464128 | 1272,215 | 2,033 | 0,042 | 0,176425 | 9,844828 |
| NA | 0,735153 | 0,737915 | 1259,241 | 0,996 | 0,319 | -0,712527 | 2,182832 |
| PCS | 2,259894 | 0,735691 | 118,760 | 3,072 | 0,003 | 0,803123 | 3,716666 |
| cohort* PCS | -2,380544 | 0,873133 | 94,899 | -2,726 | 0,008 | -4,113956 | -0,647133 |
| PA * PCS | -0,399580 | 0,095202 | 1226,025 | -4,197 | 0,000 | -0,586357 | -0,212802 |
| Cohort * PA | -6,701476 | 2,947591 | 1231,842 | -2,274 | 0,023 | -12,484330 | -0,918621 |
| Cohort * PA * PCS | 0,362487 | 0,109082 | 1199,609 | 3,323 | 0,001 | 0,148473 | 0,576501 |
| a. Dependent Variable: pain_perc. | | | | | | | |
